# Human Endogenous Retroviruses and Their Putative Role in the Development of Autoimmune Disorders Such as Multiple Sclerosis

**DOI:** 10.3389/fmicb.2018.00265

**Published:** 2018-02-20

**Authors:** Victoria Gröger, Holger Cynis

**Affiliations:** Department of Drug Design and Target Validation, Fraunhofer Institute for Cell Therapy and Immunology, Halle, Germany

**Keywords:** HERV, immune system, autoimmunity, superantigen, disease

## Abstract

Human endogenous retroviruses (HERVs) are remnants of retroviral germ line infections of human ancestors and make up ~8% of the human genome. Under physiological conditions, these elements are frequently inactive or non-functional due to deactivating mutations and epigenetic control. However, they can be reactivated under certain pathological conditions and produce viral transcripts and proteins. Several disorders, like multiple sclerosis or amyotrophic lateral sclerosis are associated with increased HERV expression. Although their detailed contribution to individual diseases has yet to be elucidated, an increasing number of studies *in vitro* and *in vivo* suggest HERVs as potent modulators of the immune system. They are able to affect the transcription of other immune-related genes, interact with pattern recognition receptors, and influence the positive and negative selection of developing thymocytes. Interestingly, HERV envelope proteins can both stimulate and suppress immune responses based on different mechanisms. In the light of HERV proteins becoming an emerging drug target for autoimmune-related disorders and cancer, we will provide an overview on recent findings of the complex interactions between HERVs and the human immune system with a focus on autoimmunity.

## Introduction

Retroelements constitute a large portion (42%) of our genome (Lander et al., 2001; Cho et al., 2008; Young et al., 2013). These transposable elements, which have RNA intermediates, are often neglected although their contribution to the human entity is not well-understood.

They are discriminated by the presence of long terminal repeats (LTRs) fundamental for regulation of retroviral gene expression (Mita and Boeke, 2016). Short interspersed nuclear elements (SINEs, without reverse transcriptase) and long interspersed nuclear elements (LINEs, with reverse transcriptase) belong to retroelements that do not possess LTRs (Mita and Boeke, 2016). LTR-positive retroelements encompass 8% of the human genome (Lander et al., 2001; Balada et al., 2009). They are either called retrotransposons or human endogenous retroviruses (HERVs) according to the absence or presence of the envelope (*env*) gene, respectively. Hence, HERVs represent the most complete form of retroelements. They entered the primate genome by exogenous retrovirus infections (Belshaw et al., 2004; Young et al., 2013). Retroviruses usually infect somatic cells, but on occasion germ line cells are also targeted. As a consequence, retroviral sequences were transmitted vertically to the offspring in a Mendelian manner and became fixed in the human population (Christensen, 2010).

HERVs are extensively distributed throughout the human genome due to amplification and transposition events. Based on sequence similarities to exogenous retroviruses, HERVs belong to class I (gamma- and epsilon-like), class II (lenti-, alpha-, beta-, and delta-like) or class III (spuma-like) retroviruses (Gifford et al., 2005; Balada et al., 2010). Phylogenetic studies revealed that at least 30 different HERV families exist in the human genome, each resulting from a distinct infection of the germ line (Bénit et al., 2003; Katzourakis et al., 2005; Stoye, 2012). Among them, HERV-K (HML-2) elements integrated most recently and thus are the most intact and biologically active forms (Marchi et al., 2014). Although the number and diversity of HERVs are huge, nomenclature is still not standardized. While most HERVs are named after the tRNA species used to prime reverse transcription (e.g., HERV-W for tryptophan tRNA), some names are still linked to the approaches applied for their identification. For more details refer to Vargiu et al. (2016).

In the course of human evolution most HERVs have accumulated mutations, which rendered a large fraction of their retroviral sequences non-functional (de Parseval and Heidmann, 2005). There are only two full-length proviruses known from the most recently integrated HERV-K family (HERV-K113, HERV-K115), which show complete reading frames for all viral genes (Turner et al., 2001). However, no infectious endogenous retrovirus has yet been identified in humans (Balada et al., 2009; Stoye, 2012). Nevertheless, intact open reading frames of single retroviral genes persisted in the genome, which gave rise to RNA transcripts as well as proteins and therefore suggesting functions in the human body (de Parseval and Heidmann, 2005).

In this regard, a well-investigated example is syncytin-1, which is an ancient Env protein from the HERV-W family. It encodes a 60 kDa large viral glycoprotein with fusogenic properties and possesses an essential function in placental development in humans (Dupressoir et al., 2012; Bolze et al., 2017). Independent integration events of syncytins, which share functional properties but are derived from multiple ERV lineages, are also important for placental development of many other mammals (Dupressoir et al., 2012; Imakawa and Nakagawa, 2017). Furthermore, HERV transcripts are upregulated during early human embryogenesis with possible implications in early viral defense pathways (Grow et al., 2015).

In surveys of the human genome, a limited number of 16 coding *env* genes were identified (de Parseval et al., 2003; Villesen et al., 2004). Although it cannot be excluded that shorter ORFs may play a role in cellular processes, it is more probable for long ORFs to have retained their original function. Consequently, the human genome bears a number of retroviral proteins with putative roles in pathophysiological conditions (Hansen et al., 2017). As an example, in amyotrophic lateral sclerosis (ALS), recent research suggested a possible involvement of HERVs (Alfahad and Nath, 2013). It was shown that HERV-K expression in human neurons causes retraction and beading of neurites (Li et al., 2015). As the virus was found to be expressed in neurons of ALS patients but not in neurons of healthy controls it was concluded that HERV-K expression might contribute to neurodegeneration (Li et al., 2015). These results are supported by findings showing increased HERV-K expression in brain tissue of ALS patients compared to non-ALS individuals (Douville et al., 2011).

The focus of the present mini-review is the putative interaction of HERV proteins with the human immune system. Different mechanisms have been proposed to explain HERV interaction with the immune response. With focus on adaptive immune mechanisms, superantigen motifs, and viral proteins will be discussed. Concerning innate immunity, interaction of HERVs with pattern recognition receptors (PRRs) like Toll-like receptor 4 (TLR4) and cluster of differentiation (CD) 14 are described. Immunosuppressive function of HERVs will be also addressed.

## Interaction of HERV proteins with the human immune system

As part of the human genome, HERV-encoded proteins should be considered as self-antigens and tolerated by the immune system. However, they could be perceived as neo-antigens if not expressed in the thymus during acquisition of immune tolerance (Balada et al., 2009). Moreover, once descended from exogenous viruses, HERVs share sequence homologies with their ancestors, which could provide antigenic epitopes for lymphocyte recognition (Voisset et al., 2008). The underlying mechanism is called molecular mimicry. Here, proteins of infectious agents such as viruses or bacteria and self-derived proteins share structural, functional or immunological similarities. In this light, sequence similarities between Env proteins of HERV-W and myelin are supposed to potentially trigger an immune response in multiple sclerosis (MS) (Ramasamy et al., 2017). There are a number of computationally predicted epitopes, which are shared between retroviruses and host proteins, although biological significance is not always given (Fujinami et al., 2006). Nevertheless, molecular mimicry could help to explain how viral infection leads to autoimmunity.

Retroviral nucleic acids and viral proteins can be sensed by a variety of PRRs, such as Toll-like receptors (TLRs) or NOD-like receptors (Thompson et al., 2011). It is conceivable that HERV-encoded proteins are able to trigger PRRs of the innate immune system leading to an induction of autoimmunity (Tugnet et al., 2013). A direct interaction between certain HERV proteins and TLRs has been shown. As an example, the surface unit of HERV-W Env binds to TLR4 and CD14 and stimulates the production of pro-inflammatory cytokines including IL-1 beta, IL-6, and TNF-alpha (Rolland et al., 2006). A more detailed description of innate immune response activation by HERVs has been compiled by Hurst et al. (Hurst and Magiorkinis, 2015).

Retroviral envelope proteins are hypothesized to both trigger and suppress an immune response. In this context, a peptide of 14 amino acids (LQARILAVERYLKD) located in the transmembrane (TM) glycoprotein gp41 of HIV-1 inhibits mitogen-induced and lymphokine-dependent T-lymphocyte proliferation (Denner et al., 1994; Mühle et al., 2017). It is also able to modulate cytokine levels as it increases IL-6 and IL-10 and decreases IL-2 and CXCL9 expression in human peripheral blood mononuclear cells (PBMCs) (Denner et al., 2013). Thereby, it allows the virus to persist and replicate in host cells (Blinov et al., 2013; Denner, 2014). This short sequence, called the immunosuppressive domain (ISD), is highly conserved among retroviruses. It was first described for murine and feline C-type retroviruses and later extended to human T-lymphotropic virus (HTLV) and HIV (Haraguchi et al., 1997). A similar but not identical sequence N-terminally to the immunodominant Cys–Cys loop can be found in some HERV families including HERV-W, HERV-FRD, and HERV-K (Morozov et al., 2013). A recombinant TM protein and a peptide corresponding to the ISD in HERV-K were shown to inhibit proliferation of human immune cells and to modulate cytokine release similar to the ISD of HIV-1 (Morozov et al., 2013), although corroboration of these findings by other groups is pending. Moreover, the envelope protein Env59 of HERV-H shows anti-inflammatory effects in an experimental arthritis model (Laska et al., 2016). In contrast to a study by Tolosa et al. showing reduced immune response of PBMCs to treatment with LPS and syncytin-1 (HERV-W) (Tolosa et al., 2012), Mangeney et al. described immunomodulatory properties for syncytin-2 (HERV-FRD) but not for syncytin-1 (Mangeney et al., 2007). However, the replacement of two amino acids in the ISD of syncytin-1 with those of syncytin-2 was able to restore the immunosuppressive function (Mangeney et al., 2007). Therefore, syncytins may help to protect the fetus from the mother's immune system (Blaise et al., 2003; Mangeney et al., 2007). HERVs might also help tumor growth by shielding it from the host immune system (Kudo-Saito et al., 2014). This was shown for a synthetic peptide corresponding to the ISD of HERV-H as it causes CCL19-mediated CD271^+^ cell-governing immunosuppression in stimulated human tumor cells (Kudo-Saito et al., 2014). HERV-H could also be an important factor for immune defense in cancer. Although the association of HERVs with cancerous tissues is beyond the scope of this review, it has been hypothesized that immune suppression by HERVs could contribute to tumor immune evasion.

## Regulation of HERV expression

HERV expression is tightly regulated by the host through epigenetic mechanisms, which results in varying expression from tissue to tissue (Hurst and Magiorkinis, 2017). Control of HERV expression depends upon regulation of the LTRs, which are able to bind nuclear transcription factors and function as promoters (Hurst and Magiorkinis, 2017). Both CpG methylation of DNA and histone deacetylation keep HERVs silenced, although histone modifications alone were shown to be insufficient for efficient transcription suppression (Hurst et al., 2016). Retroviral genes are heavily methylated in normal tissues, whereas tumors show increased levels of HERV transcripts due to hypomethylation (Cegolon et al., 2013). In addition to epigenetic regulation, other factors including hormones, microorganisms, and the environment were shown to modulate HERV expression (Balada et al., 2009; Emmer et al., 2014).

In this regard, the Epstein-Barr virus (EBV) is able to transactivate the expression of the normally inactive HERV-K18 Env protein, e.g., in resting B lymphocytes via CD21 receptor interaction (Sutkowski et al., 2001; Hsiao et al., 2006; Balada et al., 2009). The mechanism of transactivation was further shown to depend on the expression of the major EBV late gene transactivator EBNA-2 (Sutkowski et al., 2004). In-depth analysis identified the EBV latent membrane protein LMP-2A as a strong candidate for the transactivation of HERV-K18 (Sutkowski et al., 2004). Furthermore, Stauffer et al. showed that interferon-α upregulates transcription of the HERV-K18 *env* gene, suggesting an indirect connection between viral infections and autoimmune disorders (Stauffer et al., 2001). This is of great interest since HERV-K18 has been reported to have superantigen activity (Sutkowski et al., 2001; Tai et al., 2006), although conflicting data are also published (Lapatschek et al., 2000; Azar and Thibodeau, 2002).

Superantigens activate B- and T-lymphocytes regardless of the specificity of their antigen receptor. They are produced by bacteria and viruses and do not need to be processed as conventional antigens for antigen presentation (Solanki et al., 2008). They bind to conserved regions of major histocompatibility complex (MHC) class II molecules outside of the classical peptide-binding groove and connect them with a subset of T-cells expressing particular T cell receptor (TCR) β chain variable region genes (Solanki et al., 2008). This is different from conventional T-cell activation where highly variable TCR α and β chains CDR3 regions are bound (Sutkowski et al., 2001). Therefore, superantigens can stimulate many subsets of T-cells expressing the same Vβ genes, followed by massive cytokine secretion (Solanki et al., 2008).

In this context, the first HERV superantigen was isolated by Conrad et al. from pancreatic islets of patients with type I diabetes (T1D) (Conrad et al., 1997). They showed that the Env protein of this new HERV initially named IDDMK_1,2_22 has properties of a Vβ7-specific superantigen. Sequence analysis revealed that IDDMK_1,2_22 corresponds to one allele of the polymorphic HERV-K18 *env* (Stauffer et al., 2001). Sutkowski et al. further showed an activation of TCR Vβ13 T cells in response to murine B cells transfected with HERV-K18 *env* gene (Sutkowski et al., 2001). Tai and colleagues found similar results for K18 Env in mice as it expands Vβ7 and Vβ13 T cells (Tai et al., 2006; Emmer et al., 2014). Although HERV-K18 Env seems to possess superantigenic properties, its contribution to pathogenesis of T1D remains unclear. Contrary to studies supporting the initial association of the putative superantigen with T1D (Kinjo et al., 2001; Marguerat et al., 2004), four independent studies challenged this hypothesis (Badenhoop et al., 1999; Jaeckel et al., 1999; Knerr et al., 1999; Muir et al., 1999). In summary, the expression of HERVs in the human body is subject to strict regulation, which can lead to an increase in HERV transcripts and proteins due to pathological alterations.

## Implications for autoimmune disorders

The diversity of as many as 80 different types of autoimmune disorders as well as their clinical resemblance often makes diagnosis difficult. It is known that many different genetic loci with small effect sizes predispose individuals to develop autoimmunity, but in addition, environmental factors play a role in triggering the immune response (Ercolini and Miller, 2009). Here, HERVs might play an important role in the homeostasis of the immune system and could be key players when it comes to development of autoimmunity.

Studies that show an association between HERVs and autoimmune diseases either rely on retroviral antigens at the site of disease or the presence of antiretroviral antibodies in the sera of patients (Herve et al., 2002; Mameli et al., 2007; Laska et al., 2012; Alfahad and Nath, 2013). It has been hypothesized that HERVs are involved in the pathogenesis of diseases characterized by dysregulated immune response, such as autoimmune diseases (Table 1). However, whether HERVs are causative or only a consequence of disease is still under debate, as the expression of HERV mRNA or proteins at the site of tissue injury alone is insufficient to prove a pathogenic role of HERVs.

**Table 1:** Summary of HERVs associated with inflammatory diseases mainly through genetic, serological, and molecular studies.

**Diagnosis**	**HERV**	**Main results**	**References**
MS	HERV-W	Meta-analysis of HERV-W viral protein and/or mRNA expression in peripheral blood, CSF, and brain of MS patients reveals an association between HERV-W and MS	Morandi et al., 2017
		Accumulated HERV-W Gag expression in axonal structures and endothelial cells of active MS lesions, HERV-W Env expression in macrophages is restricted to early MS lesions	Perron et al., 2005
		HERV-W Env is upregulated within MS plaques and correlated with the extent of active demyelination and inflammation, significantly greater accumulation of HERV-W-specific RNAs in MS brains vs. controls	Mameli et al., 2007
		HERV-W Env is dominantly expressed in macrophages and microglia in areas of active demyelination	van Horssen et al., 2016
		MSRV *env* is significantly increased in PBMC of MS patients	Perron et al., 2012; Garcia-Montojo et al., 2013
		HERV-W Env is present in macrophages within MS brain lesions with particular concentrations around vascular elements, elevated DNA copy numbers in MS patients vs. controls	Perron et al., 2012
	HERV-H	Higher antibody reactivity toward HERV-H Env and significantly higher expression of HERV-H Env epitopes on B cells and monocytes in patients with active MS	Brudek et al., 2009
	HERV-K18	Increase in MS risk among homozygous carriers of the K18.3 allele in an US American cohort	Tai et al., 2008
		HERV-K18.3 haplotype is associated with MS susceptibility in a Spanish cohort	de la Hera et al., 2013
	HERV-Fc1	Significant increase of HERV-Fc1 RNA in plasma, and HERV-H/F Gag in T cells and monocytes of patients with active MS compared to controls	Laska et al., 2012
		Association of the HERV-Fc1 polymorphism rs391745 with bout-onset MS susceptibility in Southern European cohorts	Hansen et al., 2011; de la Hera et al., 2014
		HERF-Fc1 SNP rs391745 and HERV-K113 SNP rs2435031 synergize in influencing the risk of MS	Nexø et al., 2015; Nexø et al., 2016
ALS	HERV-K	Increased HERV-K *pol* transcripts in brain tissue of ALS patients, HERV-K expression correlates with TDP-43	Douville et al., 2011
		HERV-K is expressed in neurons of ALS patients, HERV-K expression is regulated by TDP-43 and causes retraction and beading of neurites in human neurons	Li et al., 2015
SLE	HERV-E	HERV-E mRNA expression is higher in lupus CD4+ T-cells vs. healthy controls, and positively correlated with SLE disease activity	Wu et al., 2015
	HRES-1	Small GTPase encoded by HRES-1 is overexpressed in lupus T-cells and contributes to mitochondrial dysfunction involved in SLE	Caza et al., 2014
		HRES-1 locus at the 1q42 chromosomal region influences development and manifestations of SLE	Pullmann et al., 2008
RA	HERV-K	Significantly higher serum autoantibodies against a peptide of HERV-K Env in RA patients vs. healthy controls	Mameli et al., 2017
		Significantly higher HERV-K viral loads in plasma samples from RA patients vs. healthy controls	Reynier et al., 2009
	HERV-K10	Enhanced expression of HERV-K10 mRNA in RA	Ejtehadi et al., 2006
		RA patients show significantly elevated levels of HERV-K Gag activity compared to controls	Freimanis et al., 2010
		Significantly elevated IgG antibody response to an HERV-K10 Gag peptide in patients with RA vs. controls	Nelson et al., 2014
SS	HERV-K113	HERV-K113 is found in 15.6% of 96 patients with SS	Moyes et al., 2005
	HIAP	Majority of patients with SS have serum antibodies to proteins of HIAP	Sander et al., 2005
JIA	HERV-K18	HERV-K18 transcript expression significantly elevated in JIA patients vs. controls	Sicat et al., 2005

*MS, Multiple sclerosis; ALS, Amyotrophic lateral sclerosis; SLE, Systemic lupus erythematosus; RA, Rheumatoid arthritis; SS, Sjögren's syndrome; JIA, Juvenile idiopathic arthritis*.

As a prominent example, the association of HERVs with MS is extensively discussed (Morandi et al., 2017). The multiple sclerosis associated retrovirus (MSRV) has been observed in leptomeningeal cells shed into cerebrospinal fluid of a patient with progressive MS (Perron et al., 1991). MSRV belongs to a then unknown HERV-W family and encodes a viral envelope protein that is physiologically expressed in microglia cells of normal brain (Perron et al., 2005). It becomes deregulated and is highly expressed in macrophages of active lesions in MS patients (Perron et al., 2005). In rat and human oligodendroglial precursor cells, HERV-W/TLR4 interaction causes both an increase in pro-inflammatory cytokines and nitrosative stress through increased release of inducible nitric oxide synthase. As a result, oligodendroglial differentiation is reduced, which might be the cause of impaired myelin repair observed in MS (Kremer et al., 2013). Antony and colleagues reported similar results for HERV-W Env expression in astrocytes as it leads to neuroinflammation and death of oligodendrocytes (Antony et al., 2004). Interestingly, treatment with specific antibodies against MSRV Env could prevent MS symptoms in a mouse model of experimental autoimmune encephalomyelitis (Perron et al., 2013). Clinical phase 2b studies with the same humanized antibody are currently under way in 12 European countries (CHANGE-MS study) with the possibility of an extension (ANGEL-MS study) for patients that have been enrolled in the CHANGE-MS study (Curtin et al., 2015; GeNeuro, 2017). These studies appear promising in terms of the development of potential novel therapies for MS.

HERV-Fc1, which has the potential to express a full-length Env product of 584 aa, and a Gag product of 470 aa might also be involved in the pathogenesis of MS (Nexø et al., 2015). Laska et al. could show an increased expression of HERV-Fc1 Gag in PBMCs and four times higher RNA levels in plasma of patients suffering from active MS compared to healthy controls (Laska et al., 2012). HERV-Fc1 is unusual among human proviruses in having only a single known integration in the genome (on the X chromosome; Nissen et al., 2012). This locus seems to be genetically associated with MS (Hansen et al., 2011; Nexø et al., 2016). Similarly, homozygous carriers of K18.3, which is one of three allelic forms of HERV-K18 Env and displaying superantigenic properties, show an increased risk for MS compared to individuals carrying two K18.2 alleles (Tai et al., 2008).

A possible mechanism of HERV action in MS is inferred from the findings of pre-active plaques in MS patients. These are clusters of activated microglia present in the absence of demyelination and infiltrating leukocytes (van der Valk and Amor, 2009). They can be detected by magnetic resonance imaging (MRI) several months before the appearance of an active lesion (Fazekas et al., 2002). Oligodendrocyte abnormalities and primary damage to myelin appear to be crucially involved (van der Valk and Amor, 2009). Based on these results and HERV expression in active MS lesions (Mameli et al., 2007; Perron et al., 2012; van Horssen et al., 2016), it is tempting to speculate that pathological alterations in MS are supported by HERV protein expression contributing to plaque formation. Further evidence for the role of HERVs in MS would improve our understanding of the etiology and provide new therapeutic insights into MS.

## Conclusion

The findings described here suggest that HERV elements may play a role in the pathogenesis of human diseases such as MS or ALS. Particularly in MS, it is conceivable that the formation of HERV Env proteins trigger a damaging cascade that eventually leads to the symptoms of the disease. This assumption could help to integrate unexpected findings, such as pre-active plaques, into the sequence of pathological events (Christensen, 2017). A deeper understanding of HERV expression under physiological and pathophysiological conditions and their interaction with the immune system might help to better explain and combine several factors that contribute to MS. In this regard, the first studies targeting a specific HERV-W Env protein are currently in clinical trials and may provide further evidence of the validity of this novel approach in the near future.

## Author contributions

VG and HC: designed the outline of the manuscript; VG: wrote the manuscript; HC: supervised the writing, edited, and approved the final version of the manuscript.

### Conflict of interest statement

The authors declare that the research was conducted in the absence of any commercial or financial relationships that could be construed as a potential conflict of interest.
